# Insecticide Resistance Profile and Mechanisms in *An. gambiae* s.l. from Ebolowa, South Cameroon

**DOI:** 10.3390/insects13121133

**Published:** 2022-12-08

**Authors:** Salomon Efa, Emmanuel Elanga-Ndille, Yacouba Poumachu, Billy Tene, Jacqueline Ze Mikande, Njoumémi Zakariaou, Charles S. Wondji, Cyrille Ndo

**Affiliations:** 1 Department of Medical Entomology, Centre for Research in Infectious Diseases (CRID), Yaoundé P.O. Box 13591, Cameroon; 2 Faculty of Sciences, University of Yaoundé I, Yaoundé P.O. Box 337, Cameroon; 3 Vector Borne Parasitic and Infectious Diseases Unit of the Laboratory of Applied Biology and Ecology (VBID-LABEA), Department of Animal Biology, Faculty of Sciences, University of Dschang, Dschang P.O. Box 067, Cameroon; 4 Department of Anesthesia and Reanimation, Faculty of Medicine and Biomedical Sciences, University of Yaoundé I, Yaoundé P.O Box 1364, Cameroon; 5 Department of Vector Biology, Liverpool School of Tropical Medicine, Pembroke Place, Liverpool L3 5QA, UK; 6 Faculty of Medicine and Pharmaceutical Sciences, University of Douala, Douala P.O. Box 2701, Cameroon

**Keywords:** insecticide resistance, carbamates, organophosphates, pyrethroids, *Anopheles gambiae* s.l., Ebolowa

## Abstract

**Simple Summary:**

Monitoring insecticide resistance can help designing strategies to delay or prevent its onset and spread in vector populations. This study aimed at evaluating levels and mechanisms responsible of insecticide resistance in the major malaria vector *Anopheles gambiae* s.l. from Ebolowa (South Cameroon). Methods: Mosquito were collected in temporary water pools as larvae and reared to adult stages, which were tested against carbamate, organophosphate and pyrethroid insecticides as recommended by the World Health Organization (WHO). The implication of DNA mutations and enzymes that allow mosquitoes to survive insecticide exposure was also investigated. Results: *Anopheles coluzzii* was the predominant (99%) species in Ebolowa and was fully sensitive to carbamates and organophosphates, but highly resistant to the pyrethroids. Pre-exposure to the piperonyl butoxide (PBO), an inhibitor of detoxification enzymes, allowed deltamethrin to recover its full efficacy, but not permethrin and alphacypermethrin. Only the L1014F (*kdr*-West) mutation was present, and at a high frequency (75%), likely causing resistance to permethrin and alphacypermethrin, but not deltamethrin. Conclusion: The increased resistance pyrethroids in *An. gambiae* s.l. from Ebolowa could jeopardize the efficacy of Long-Lasting Insecticidal Nets (LLINs) used for malaria vector control. There is an urgent need to put in place resistance management strategies in this locality.

**Abstract:**

Monitoring the trend of insecticide resistance and understanding associated genetic mechanisms is important for designing efficient malaria vector control strategies. This study was conducted to provide temporal data on insecticide resistance status and mechanisms in the major malaria vector *Anopheles gambiae* s.l. from Ebolowa, Southern Cameroon. Methods: Larvae of *An. gambiae* s.l. were collected from typical breeding sites throughout the city and reared to adulthood. Emerging adults were morphologically identified and WHO tube assays were performed to determine their susceptibility to carbamate, organophosphate and pyrethroid insecticides at diagnostic doses. When resistance was observed, its intensity was determined by performing WHO tube tests using 5 and 10 times the concentration of the diagnostic dose. Metabolic resistance mechanisms were investigated using insecticide-synergist assays. Sibling species of the *An. gambiae* complex were identified using SINE-PCR protocol. TaqMan assay was used to genotype the L1014F and L1014S *kdr* mutations, and the N1575Y mutation, an amplifier of the resistance conferred by the L1014F mutation. Results: *Anopheles coluzzii* was by far the dominant (99%) member of the *An. gambiae* s.l. complex in Ebolowa. The species was fully susceptible to carbamates and organophosphates, but resistant to all pyrethroid insecticides tested. Resistance was of moderate intensity for deltamethrin (mortality: 37%, 70% and 99% for 1×, 5× and 10× insecticide concentration, respectively) but rather of high intensity for permethrin (5% for 1×; 62% for 5× and 75% for 10×) and for alphacypermethrin (4.4% for 1×; 57% for 5× and 80% for 10×). Pre-exposure to the synergist PBO resulted in a full recovery of the susceptibility to delthametrin, but this was not observed for the other two pyrethroids tested. L1014S (*kdr*-East) and the N1575Y mutations were absent, whereas the L1014F (*kdr*-West) mutation was present at a high frequency (75%), showing a significant association with resistance to permethrin (OR = 3.8; 95%; CI [1.9–7.4]; *p* < 0.0001) and alphacypermethrin (OR = 3; 95%; CI [1.6–5.4]; *p* = 0.0002). Conclusion: The increased resistance of *An. gambiae* s.l. to pyrethroid insecticides as observed in Ebolowa poses a threat to the efficacy of LLINs used to protect populations from the bites of *Anopheles* mosquitoes that transmit malaria parasites. The present study further highlights the urgent need to implement resistance management strategies in order to maintain the effectiveness of insecticide-based vector control interventions and prevent a rebound in malaria-related mortality.

## 1. Introduction

Sub-Saharan Africa is the host bed of malaria. In 2020, this region accounted for 95% of world morbidity and mortality, with an estimated 228 million malaria cases and 602,000 malaria deaths [1]. Vector control represents a central and critical component of malaria control strategies. It primarily relies on two insecticide-based interventions including Long-Lasting Insecticidal Nets (LLINs) and indoor residual spraying (IRS) of insecticides. World Health Organization (WHO) recommends five main classes of insecticides for IRS including pyrethroids, organochlorines, organophosphates, carbamates and neonicotinoids which were recently added [2,3]. Of these insecticides classes, only pyrethroids are used for bed nets impregnation [4].

Estimates showed that current coverage in LLINs and IRS in the sub-Saharan Africa averts approximately 220,000 annual deaths among children under five years [4]. In Cameroon, which accounts among the 13 countries with the highest malaria burden worldwide, over 35 million LLINs have been freely distributed to populations since 2000 [5]. This scale-up of LLINs resulted in significant decrease in national malaria prevalence from 41% to 24.3%, between 2000 and 2015, and a 54% reduction in malaria–associated deaths (from 13,000 to 6000) [5]. An IRS program, which will be conducted by the VectorLink project under the sponsorship of the US President Malaria Initiative (PMI), is in its preparatory phase [5,6].

The increased use of insecticides in public health and pesticides in agriculture has unfortunately selected insect vectors with the ability to survive exposure to insecticides owing to physiological or behavioural adaptations [7,8]. Currently, this resistance has rapidly spread across Africa and is considered as a potential threat to malaria control [1]. In Cameroon, resistance in *An. gambiae* s.l. was reported for insecticide classes used for vector control except organophosphates [9]. High resistance levels to pyrethroids (permethrin, deltamethrin and alphacypermethrin) and to organochlorines (DDT) were reported in almost all study sites across the country. This resistance was mainly associated with high frequencies of *kdr*-West (L1014F) resistant allele and overexpression of detoxification genes belonging to the cytochrome P450 family [9]. Resistance to carbamates (bendiocarb) was observed in a few sites and was attributed to overexpression of several P450 genes [10,11,12]. Recently, the Acetylcholinesterase (ace-1R) target site mutation G119S conferring resistance to carbamates and organophosphates was detected for the first time in *An. gambiae* s.l. populations of South and Centre regions [13,14,15].

Widespread failure of the insecticides, especially pyrethroids, could lead to devastating consequences, since much of the progress achieved in reducing the burden of malaria through vector control would be lost [1,16]. The development and implementation of resistance management plan, that aims to deploy available interventions in most efficient way to maintain effective malaria control, is therefore a pressing issue. The WHO further recommended that routine monitoring of vector susceptibility to the insecticides be conducted in order to establish contemporary baseline data that can serve as evidences for accurate decision-making [16,17]. In Cameroon, data on insecticide resistance in malaria vectors are still lacking in several localities. This, for example is the case of Ebolowa, a fast growing and urbanising city of South Cameroon, which is becoming an important educational and industrial zone, with the recent installation of a University and the construction of a deep-sea port in a neighbouring city (Kribi).

In the current study, WHO tube tests were performed to assess the profile and intensity of insecticide resistance in *An. gambiae* s.l. population from Ebolowa. Additional molecular (polymerase chain reaction, PCR) analysis were conducted to determine underlying genetic mechanisms. Data generated will help the National Malaria Control Program (NMCP) to better plan vector control in this locality and to anticipate modification in malaria epidemiology, especially vector biology, that may occur due to ongoing environmental and demographic changes.

## 2. Methods

### 2.1. Study Site

**Figure 1:**
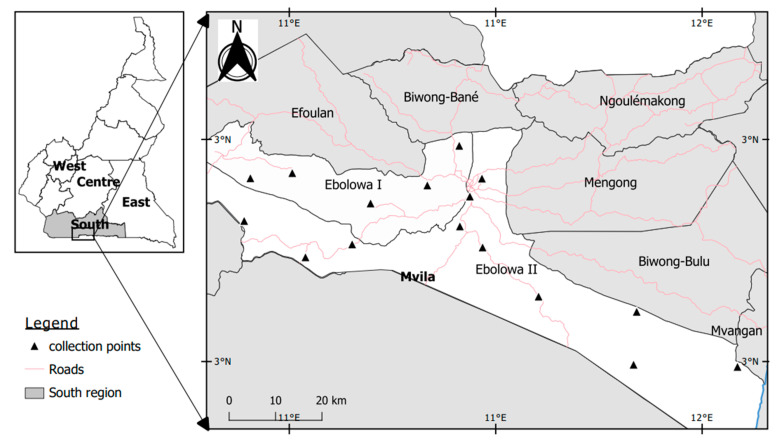
MAP of Cameroon showing the geographical location of EBOLOWA and distribution of larval collection points.

Ebolowa (2°54′ N, 11°9′ E) is situated in the heart of the equatorial forest of the South region, and about 168 Km from Yaoundé the Capital city of Cameroon (Figure 1). This locality of 56 Km^2^ is populated of about 250,000 inhabitants. The climate is of Equatorial-Guinean type, with average annual rainfall between 1400 mm and 2500 mm. Precipitations are frequent throughout the year, with the maximum observed in September–October and the minimum in December–January. The average annual temperature is around 25 °C [18].

### 2.2. Mosquito Larval Collection and Rearing

The study was conducted between September and October 2021 during the wet rainy season. Mosquito larvae were collected in temporary sunny water bodies throughout the city using deeping method (Figure 1) [19]. Larvae were transferred into labelled plastic containers and transported to the insectary, where they were reared to adult stages under controlled conditions (25 ± 2 °C temperature, 70–80% relative humidity, 12:12-h light/dark cycle). Emerged adult mosquitoes were identified as *An. gambiae* s.l. based on morphological criteria and fed with 10% sucrose solution until tests were performed.

### 2.3. Insecticide Susceptibility Test

The susceptibility of field *An. gambiae* s.l. population to three major classes of insecticides used in public health was assessed including pyrethroids (0.75% permethrin, 0.05% deltamethrin, 0.25% alphacypermethrin), carbamates (0.1% bendiocarb, 0.1% propoxur) and organophosphates (0.25% pirimiphos methyl, 1% fenitrothion) [20]. Whatman papers impregnated with WHO discriminating dosages of the insecticides were purchased from the Vector Control Research Unit, University of Sains, Malaysia. Insecticide impregnated papers were first tested against *An. gambiae* Kisumu reference susceptible strain to ensure they were of good quality.

Bioassays were performed following WHO tubes test protocol [20]. Each test consisted of four replicates (tubes) of 20–25 female mosquitoes aged between 2 and 5 days that were exposed to insecticide-impregnated papers for one hour. The control consisted of two replicates of the same number of mosquitoes that were exposed in parallel to non-impregnated papers for the same duration. At the end of the 60 min exposition period, the number of mosquitoes knocked down was recorded in each tube. Test mosquitoes were gently transferred to the holding-tubes (lined with non-impregnated papers) and supplied with 10% sugar solution. Mortality was determined 24 h post-exposure to insecticides with dead and alive mosquitoes separately kept in desiccant [20]. When resistance was detected, its intensity was assessed by exposing mosquitoes to 5 and 10 (when resistance was also observed at 5×) times the concentration of the diagnostic dose of the insecticide as described above.

### 2.4. Synergist Bioassays

The presence of metabolic resistance mechanisms in *An. gambiae* s.l. from Ebolowa was assessed using tests that combined pyrethroids with 4% piperonyl butoxide (PBO) synergist, an inhibitor of P450s mono-oxygenases, which play important role in metabolizing these insecticides [20]. Tests were performed as described above with the exception that mosquitoes were pre-exposed to PBO-impregnated papers for one hour before exposure to the insecticides (0.75% permethrin, 0.05% deltamethrin or 0.25% alphacypermethrin) for an additional hour. In addition, control assays using only PBO-impregnated papers were also performed. Similarly, the number of mosquitoes knocked down after one hour of exposition to the insecticides was recorded. Mortality was evaluated after 24 h and mosquitoes alive and dead were separately stored in tube containing silica gel (desiccant) and kept at −20 °C for molecular analysis.

### 2.5. Molecular Species Identification and Detection of Resistance-Associated Mutations

Genomic DNA was extracted from individual alive and dead mosquitoes from bioassays using the Livak protocol [21]. Sibling species of the *An. gambiae* complex were identified according to the SINE-PCR molecular method described by Santolamazza et al. [22]. This PCR is based on the amplification of a nearly 200 bp long Short Interpersed Element (SINE 200) locus on chromosome X (locus S200X6.1.) using the primers SINE 200X6.1F (5′-TCG CCT TAG ACC TTG GGT TA-3′) and SINE 200X6.1R (5′-CGC TTC AAG AAT TCG AGA TAC-3′). The amplified fragment length is 479 pb in *An. coluzzii*, for which the insertion is fixed and 249 bp in *An. gambiae* due to the absence of the insertion. The TaqMan real-time PCR assay, described by Bass et al. [23], was used to genotype the *kdr* mutations (L1014F, L1014S), and the N1575Y mutation which amplifies resistance conferred by L1014F [24].

### 2.6. Data Analysis

Knock-down rate was calculated by dividing the number of individuals immobilized at the bottom of the tubes one hour after insecticide exposure by the total number of mosquitoes tested. Mortality rates were calculated by dividing number of dead individuals by the total number of mosquitoes tested. Since no mortality greater than 5% was recorded in the control tests, the Abbott’s correction was not applied.

For bioassays performed with discriminating concentrations (1×) of the insecticides to determine phenotypic resistance, mosquitoes were considered as susceptible when mortality was ≥98%, probable resistant for mortality ranged between 90 and 97%, and resistant when it was <90%.

The following interpretation parameters were used for bioassays performed to assess resistance intensity:

mortality ≥ 98% at 5× doses indicated that the resistance observed is of low intensity and no further test at 10× doses was required;

mortality < 98% at 5× doses indicated that resistance is of moderate to high intensity that was further assessed at 10× doses;

mortality ≥ 98% at 10× doses confirmed the moderate intensity suspected at 5× doses, while mortality < 98% was indicative of high resistance intensity.

Regarding synergist bioassays, metabolic mechanisms were considered not involved when synergist-insecticide mortality rate was not higher than for insecticide-only. When synergist-insecticide mortality was <98% but higher than for insecticide-only, metabolic mechanisms were partially involved. When synergist-insecticide mortality was ≥98% and higher than for insecticide-only, metabolic mechanisms were fully involved [20].

Allelic frequencies were calculated using the formula ƒ(R) = (2n. RR + n.RS)/2N, where n is the number of mosquitoes of a given genotype, and N is the total number of mosquitoes analysed. The correlation between *kdr* genotypes and resistance phenotypes was assessed by estimating the odds ratios and the statistical significance based on the Fisher exact probability test. Statistical analyses were performed using VassarStats software packages online. GraphPad Prism version 7.00 (GraphPad Software Inc., La San Diego, CA, USA) was used to construct the graphs.

## 3. Results

### 3.1. Resistance Profile of An. gambiae s.l. to the Insecticides in Ebolowa

One hundred percent (100%) mortality was recorded for the *An. gambiae* Kisumu susceptible reference strain, confirm the insecticide-treated papers used were of good quality. According to the standard WHO criteria, *An. gambiae* s.l. from Ebolowa was susceptible to carbamates with mortality rates of 98.81% for bendiocarb and 100% for propoxur. A complete susceptibility to pyrimiphos-methyl and fenitrothion (organophosphates) was also observed, with 100% mortality rates. In contrast, resistance was observed for all the pyrethroids tested, with mortality rates of 37% for deltamethrin, 5% for permethrin and 4.44% for alphacypermethrin (Table 1).

### 3.2. Intensity of Resistance to Pyrethroids

**Figure 2:**
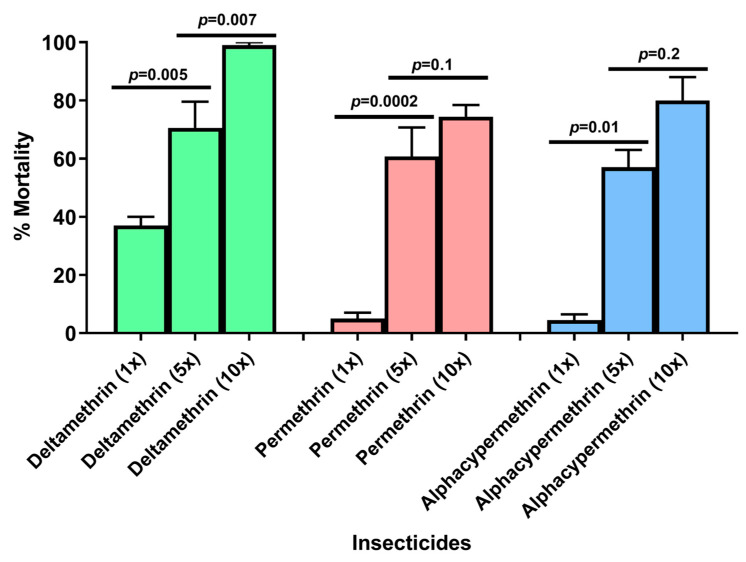
**Evolution of the mortality rate according to the concentration of the different insecticides.** (The error bars represent the standard error of the mean (SEM) of mortality rate. Green: mortality rate at different doses of deltamethrin; red: mortality rate at different doses of permethrin; blue: mortality rate at different doses of alphacypermethrin).

Bioassays performed using 5 times and 10 times the diagnostic concentrations of pyrethroid insecticides gave contrasting results. For deltamethrin, mortality rates were 70% and 99% for 5× and 10× doses, respectively, indicating moderate resistance intensity. In contrast, resistance intensity was high for the two other pyrethroids. For permethrin, mortality rates were 62% and 75% for 5× and 10× doses, respectively. For alphacypermethrin, mortality rates were 57% for 5× dose and 80% for 10× **(**Figure 2).

### 3.3. Synergist Bioassays

**Figure 3:**
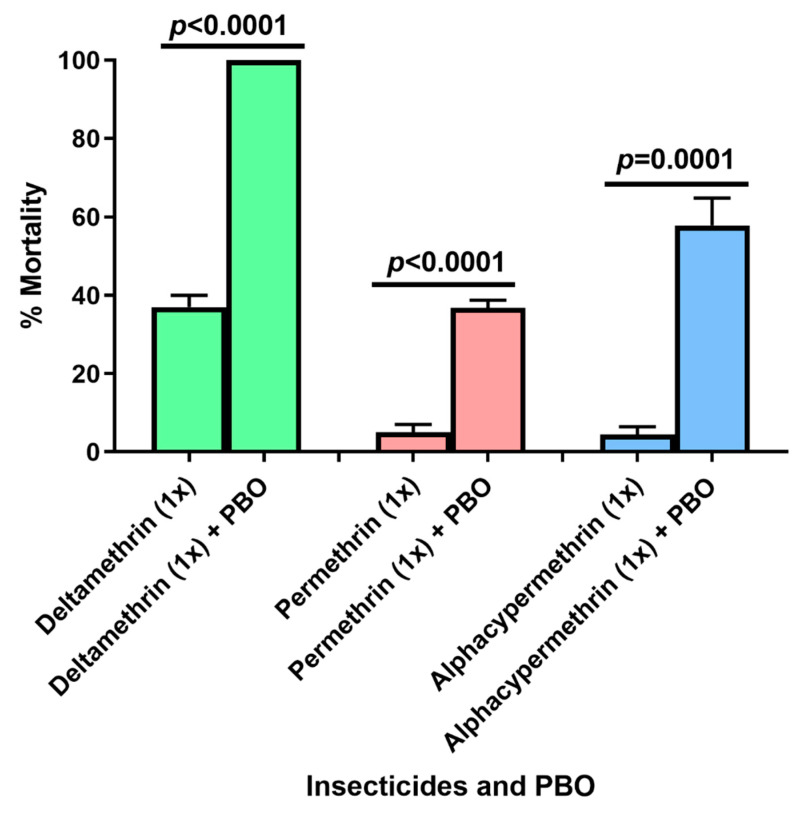
**Mortality rate induced by the different pyrethroids used alone or with the addition of PBO.** (The error bars represent the standard error of the mean (SEM) of mortality rate. Green: mortality rate at different doses of deltamethrin + PBO; red: mortality rate at different doses of permethrin + PBO; blue: mortality rate at different doses of alphacypermethrin + PBO).

Results of PBO-insecticides bioassays revealed different level of implication of cytochrome P450 mono-oxygenases in *An. gambiae* s.l. resistance against pyrethroids in Ebolowa. Pre-exposure of mosquitoes to PBO led to a full recovery of susceptibility (100% mortality) to deltamethrin, whereas only a partial recovery of susceptibility was observed for the two other pyrethroid insecticides. For permethrin, mortality increased from 5% (for permethrin 1×) to 37% (for permethrin 1× + PBO), while for alpha-cypermethrin, it increased from 4% (for alphacypermethrin 1×) to 58% (for alphacypermethrin 1× + PBO) (Figure 3).

### 3.4. Molecular Identification of Species of the An. gambiae Complex and Genotyping of Mutations Driving Insecticide Resistance

Genomic DNA was extracted from a subsample of 96 mosquitoes used for bioassays using permethrin (dead = 5; alive = 27); deltamethrin (dead = 12; alive = 20); and alphacypermethrin (dead = 5; alive = 27). The SINE-PCR yield was 96% revealing the following species composition: 99% of *An. coluzzii* and 1% of *An. gambiae*.

**Figure 4:**
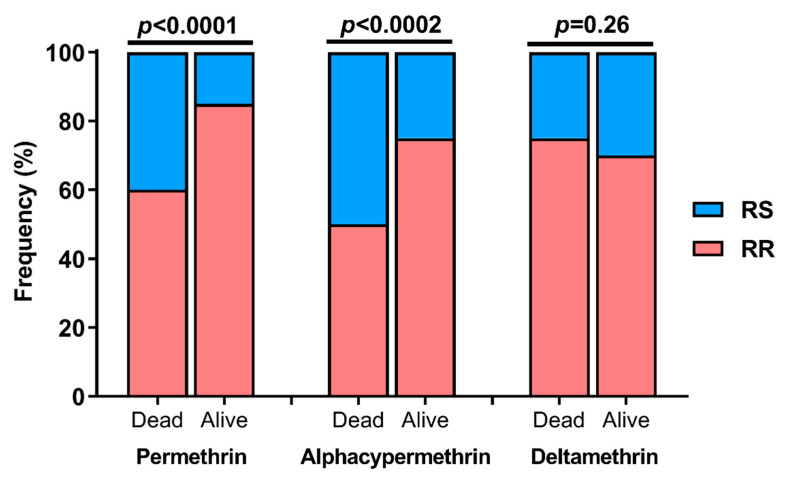
**Level of association between the L1014F (*kdr*-West) mutation and pyrethroids resistance** (RR: homozygous resistant genotype; RS: heterozygous genotype).

In addition, these 96 samples were genotyped for the L1014F (*kdr*-West), L1014S (*kdr*-East) and N1575Y mutations. The L1014S (*kdr*-East) and N1575Y mutations were absent, whereas the L1014F (*kdr*-West) mutation was present at a high frequency (75%). L1014F genotypes included 50% of resistant homozygous (RR) and 50% of heterozygous (RS). For tests performed with permethrin and alphacypermethrin, the frequency of the L1014F (*kdr*-West) was significantly higher in alive than in dead mosquitoes. This trend was not observed in test done with deltamethrin (Figure 4). Consequently, mosquitoes carrying the L1014F mutation were likely to survive exposure to permethrin (OR = 3.8; 95% CI [1.9–7.4]; *p* < 0.0001) and alphacypermethrin (OR = 3; 95% CI [1.6–5.4]; *p* = 0.0002). In contrast, this association was not observed for deltamethrin (OR = 0.7; 95% CI [0.4–1.4]; *p* = 0, 26).

## 4. Discussion

The ubiquitous use of pyrethroids for treatment of nets and the long use of other classes, such as organochlorine, carbamate and organophosphate compounds, for IRS are likely to have contributed to the development, spread and increase in resistance among malaria vectors. Given the desire to preserve recent gains made in malaria control, by implementing resistance management strategies, routine monitoring of insecticide resistance and of the mechanisms involved in major malaria vector across endemic countries is highly recommend by the WHO.

*Anopheles coluzzii* is one of the major malaria vectors in sub-Saharan Africa and was the predominant member species of *An. gambiae* complex in Ebolowa, likely due to its capability to tolerate organic pollutants that are usually present in urban breeding sites [25,26]. In Ebolowa, this species exhibited resistance to all pyrethroid insecticides tested. The intensity of this resistance varied according to the insecticide and it was high for permethrin and alphacypermethrin, but moderate for deltamethrin. These results are similar to those reported in neighbouring localities of Sangmelima and Nyabessan, indicating that resistance to pyrethroids in *An. gambiae* s.l. may now be generalized in South Cameroon [14]. Moreover, the resistance profile to pyrethroids observed in this study is in line with the escalation of insecticide resistance recently reported across Cameroon and several other countries of sub-Saharan Africa [9,13,15,27,28,29,30,31]. This situation is of concern to malaria researchers and decision-makers, as pyrethroids remain the only insecticide class recommended by the WHO for bed net impregnation [20]. There is a fear that pyrethroids resistance could lead to a drop in efficacy of LLINs, thus alleviating the gains in term of reduction in malaria morbidity and mortality. Nevertheless, the epidemiological impact of insecticide resistance remains a matter of debate. Laboratory studies showed limited efficacy of several brands of LLINs against pyrethroid resistant *An. gambiae* s.l. and *An. funestus* field populations across several countries of Central, West, East and South Africa [27,30,31,32,33,34]. By contrast, field trials conducted in experimental huts showed that LLINs, particularly those incorporating a synergist (PBO), still perform well by inhibiting mosquito blood feeding on human, or by preventing them from entering the houses (deterrence) [35,36,37]. These contrasting reports therefore call for more studies to unravel the epidemiological impact of insecticide resistance.

Whatever the above opinions (whether insecticide resistance impact or not the efficacy of LLINs) may be, specific actions are in dire need to manage insecticide resistance and to preserve efficacy of the limited arsenal of the insecticides available for public health interventions. The design of strategies for the management of insecticide resistance requires a good understanding of mechanisms involved [17]. Results of the present study suggest that cytochrome P450 was fully involved in resistance to deltamethrin in *An. gambiae* s.l. from Ebolowa, while a combination of metabolic (P450) and molecular (L1014F mutation) mechanisms conferred resistance to permethrin and alphacypermethrin. Although the sample size used for *kdr* analysis was small, this pattern is consistent with reports from several regions across sub-Saharan Africa highlighting the complexity of insecticide resistance phenomenon [38,39,40].

To curb insecticide resistance, the use of pyrethroids only-based nets should be avoided in areas where pyrethroid resistance already emerged. In such areas, distribution of PBO-pyrethroids nets (PBO-deltamethrin nets in the case of Ebolowa) or new generation nets, such as Interceptor G2, that combines the pyrrole chlorfenapyr and alphacypermethrin, should be prioritized. In addition, indoor residual spray intervention using carbamates or organophosphates can be implemented where vector populations remain susceptible to these insecticide classes, as it is the case in Ebolowa. Moreover, efforts should be made to reduce the use of insecticides in public health and agricultural activities. Nonchemical control measures should be developed, strengthened, diversified and widely adopted. This should include, for example, housing improvement, sterile insect technique, attractive sugar baits and eave tubes. These non-chemical control methods could also contribute controlling malaria in areas where bed nets are poorly used or where mosquitoes are exophagic.

## 5. Conclusions

The present study aimed at assessing insecticide resistance profile and the underlying mechanisms in *An. gambiae* s.l. population from Ebolowa (South Cameroon). The species exhibited full susceptibility to organophosphates and carbamates, but high resistance to pyrethroid insecticides. Cytochrome P450 was fully involved in resistance to deltamethrin, whereas resistance against permethrin and alphacypermethrin was driven by a combination of metabolic (P450) and molecular (L1014F mutation) mechanisms. Results of this study further confirm escalation of insecticide resistance in malaria vectors and this once more rings the alarm bell for rapid design and implementation of resistance management strategies.

## Figures and Tables

**Table 1:** Number of mosquitoes tested and mortality rates in *An. gambiae* s.l. fro Ebolowa following insecticide bioassays.

Insecticides Classes	Insecticides Tested (Concentration)	Number Tested	TKD60 (IC à 95%)	MR (%)	Status
Pyrethroïds	deltamethrin (0.05)	100	40.0 (0.309–0498)	37	R
	permethrin (0.75)	100	1 (0.002–0.054)	5	R
	alphacypermethrin (0.05)	100	24.4 (0.167–0.332)	4.4	R
Carbamates	bendiocarb (0.1)	84	100 (0.956–1)	98.8	S
	propoxur (1)	84	100 (0.956–1)	100	S
Organophosphates	pyrimiphos-methyl (0.25)	86	89.5 (0.678–0.642)	100	S
	fenitrothion (0.25)	84	100 (0.956–1)	100	S

R = resistant; S = Susceptible; TKD60 = knock down at 60 min; MR= Mortality rate.

## Data Availability

The datasets generated and/or analysed during the current study are available from the corresponding author on reasonable request.
